# Dual-Band Resonant Acoustic Metasurfaces from Nested Negative Effective Parameter Unit

**DOI:** 10.3390/ma18122811

**Published:** 2025-06-15

**Authors:** Limei Hao, Dongan Liu, Xiaole Yan, Qingning Yang, Jifeng Guo, Xingchen Tian, You Xie, Shaofang Pang, Tao Zhang, Zhi Chen

**Affiliations:** 1 College of Science, Xi’an University of Science and Technology, Xi’an 710054, China; 22201104021@stu.xust.edu.cn (D.L.); yanxl@xust.edu.cn (X.Y.); 23201223074@stu.xust.edu.cn (Q.Y.); 22201104031@stu.xust.edu.cn (J.G.); 23201223075@stu.xust.edu.cn (X.T.); xieyou@hotmail.com (Y.X.); pangshaofang814@hotmail.com (S.P.); tzhang@xust.edu.cn (T.Z.); 2 Department of Applied Physics, Northwestern Polytechnical University, Xi’an 710129, China

**Keywords:** resonant metasurface, negative effective mass density, negative effective modulus, nested structure

## Abstract

Phase gradient acoustic metasurfaces often exhibit pronounced structural dependence in imaging applications, with significant performance variations arising from differences in the negative effective parameters of resonant unit cells. However, the relationship between imaging performance and negative effective parameters near resonance frequencies—particularly in multi-band nested structures—remains insufficiently studied. To address this knowledge gap, this work combines effective parameter theory with local resonance characteristics to construct a comparative model investigating how negative effective mass density and modulus influence the imaging quality of single-band and dual-band nested metasurfaces in series and parallel configurations. The results demonstrate that (1) for single-band structures, imaging performance positively correlates with the absolute value of negative effective parameters; (2) in dual-band configurations, smaller inter-band differences in negative parameter values yield more stable imaging; and (3) series-type nested structures exhibit superior reflection imaging performance compared to parallel-type structures, though with marginally reduced design flexibility. This study elucidates the fundamental mechanisms through which negative parameters govern acoustic metasurface imaging and provides theoretical foundations for designing multi-band acoustic devices.

## 1. Introduction

The effective modulation of sound waves represents a pivotal area of scientific inquiry. Despite the fact that the operational scales of acoustic metasurfaces are generally substantial, their fundamental physical mechanisms, experimental validation values, fabrication advantages, and application potentials continue to render them significant in the realm of nanometer-scale research. A thorough investigation into the fluctuation modulation principle of metamaterials at the micrometer/millimeter scale can provide theoretical support and technical reference for future device development at the nanometer scale. In recent decades, there has been a clear and growing demand for more effective acoustic solutions, including noise cancelation and reduction, which has resulted in significant advancements in the field of acoustic metasurfaces (AMSs) over the past two decades. Metasurfaces [1,2], as a type of two-dimensional metamaterial [3,4], have a wide range of potential applications in the field of miniaturization [5,6,7,8,9]. Zhu et al. proposed an ultrathin Schröder diffuser based on AMSs by utilizing the concept of negative Young’s modulus structure modulation on the phase, achieving an enhanced acoustic diffuse reflection and an improved acoustic diffusion effect. Such techniques enable enhanced acoustic diffusion effects [10] and perfect absorbers [11,12,13] at lower frequencies, as well as stealth and focusing [14,15,16,17,18]. Moreover, the 2π variation in the phase can be regulated by modifying the following: the width and thickness of the thin-film structural units with negative mass properties; the Young’s modulus [19] and mass density [20] of the material; and parameters such as the film tension, the shape, and the mass of the mass block. This approach can thereby achieve acoustic phenomena including anomalous reflections, focusing, and acoustic cloak stealth [21,22,23,24,25,26,27].

A substantial body of research has been dedicated to the field of metasurfaces for acoustic wave modulation. However, in the realm of optical metasurfaces, the modulation function is predominantly realized through the design of specific structural units [2,28,29,30,31,32]. The parallel connection of several Helmholtz resonators with differing resonance frequencies enables the generation of anomalous reflection, acoustic focusing, diffuse reflection, and other effects across multiple frequency bands [10,16,33,34]. The combination of two units with negative modulus and mass characteristics has been demonstrated to achieve even more effective modulation [35,36]. The structural units exhibit excellent acoustic properties, and there is no shortage of metamaterials with these characteristics. Nevertheless, no article has yet been published from this perspective. However, the findings of our present study demonstrate that (1) not all resonance units are capable of achieving optimal imaging outcomes; (2) the imaging performance of the metasurface designed with non-negative parameter structural units is not as good as that of the metasurface designed with negative parameter structural units; and (3) the imaging effect is unstable.

In this work, in contrast to the earlier studies, our design utilizes acoustic unit cells with negative effective parameters, enabling enhanced energy accumulation and improved imaging performance. Furthermore, by introducing nested structures, we achieve improved tunability in negative modulus-based designs, albeit with slightly reduced imaging quality. We adopt resonance-type metasurfaces, following the methodology outlined in reference [3]. The negative effective mass density and negative effective modulus serve as the basis for designing two fundamental units: the single-open-hole hollow circular tube and the single-layer cube. Through nesting configurations, these units are employed in the active design of dual-band metasurfaces based on resonant frequencies. The application of electromagnetic waves is realized through geometrical scaling, material optimization, and manufacturing process adaptation.

## 2. Model and Methods

### 2.1. Model

The methodology utilizes a nested Split Hollow Cuboid (NSHC) model with a negative effective modulus and a nested single-opening hollow tube (NSHT) model with a negative effective mass density [37].

**Figure 1:**
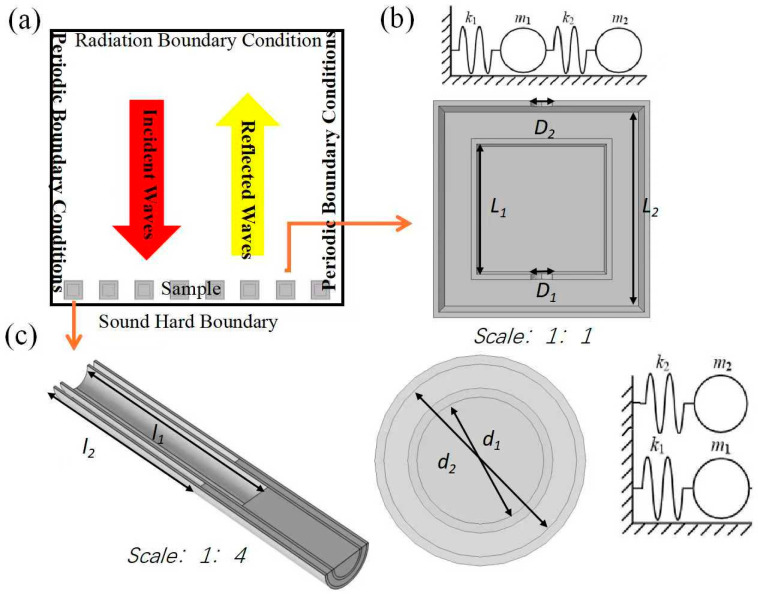
(**a**) Diagram of the acoustic metasurfaces (AMSs) with periodic boundaries in the simulation environment, (**b**) section diagram and equivalent circuit diagram of a nested structure unit with a negative effective modulus, and (**c**) section diagram and equivalent circuit diagram of a nested structure unit with a negative effective mass.

The design and simulation of the AMSs are conducted using a 3D acoustic model in COMSOL 5.6 multiphysics with the finite element method (FEM). The detailed simulation condition settings and the three-dimensional view of the NSHC and NSHT are presented in Figure 1. In the numerical simulation, the AMSs are positioned within a waveguide. The four sides of the waveguide are designated as periodic boundary conditions; the top surface represents the incident surface with a plane wave radiation condition of 1 Pa; and the bottom surface is defined as a hard boundary condition. The sample is established as a solid domain, and the material utilized is PLA (Polylactic Acid), a 3D printing material with a density of 1000 kg/m^3^ and a modulus of elasticity of 3 × 10^9^ Pa. The entire simulation environment is designated as the fluid domain, with air serving as the material, exhibiting a speed of sound of 343 m/s and a density of 1.29 kg/m^3^.

During the meshing process, the two adjacent faces are meshed using a free triangular mesh. Subsequently, the mesh of each face is replicated to the opposing face. Finally, the whole simulation domain is meshed using a face-coupled tetrahedron, with a maximum element size of (1300(m/s)/fmax)/9 for the fluid domain and (343(m/s)/fmax)/6 for the solid domain. The total number of mesh elements used in our model is approximately 220,000. The simulation runs on a high-performance computing server with dual Intel Xeon 8275CL processors (32 cores, 64 threads) and 512 GB RAM.

The center distances between neighboring resonance cells are defined as the AMS period (T). The phase gradient of the AMSs is closely related to the period, which is calculated as π/4T. The NSHC model with a negative effective modulus is configured with inner and outer side lengths of 13 mm and 20 mm, respectively, and inner and outer aperture diameters of 3 mm. The wall thickness is 0.5 mm. The NSHT model with a negative effective mass density has the following dimensions: inner tube length (*l*_1_) = 90 mm, outer tube length (*l*_2_) = 80 mm, inner tube diameter (*d*_1_) = 7.00 mm, outer tube diameter (*d*_2_) = 10.35 mm, and wall thickness = 1.00 mm, as shown in Figure 1.

### 2.2. Theoretical Analysis

A schematic of the NSHC model with a negative effective modulus is presented in Figure 1b. Based on the principle of acoustic force analogy, its structure can be equated to a tandem-type double-spring vibrator system, in which the cavity is equivalent to a spring (designated as “*k*”) and the small hole is equivalent to a mass (designated as “*m*”). The two resonant frequencies of the resonant structure are as follows:

(1) $$f_{1}=\frac{\omega_{1}}{2\pi }=\frac{1}{2\pi }\sqrt{\frac{b+\sqrt{b^{2}-4a}}{2}},$$

(2) $$f_{2}=\frac{\omega_{2}}{2\pi }=\frac{1}{2\pi }\sqrt{\frac{b-\sqrt{b^{2}-4a}}{2}},$$

where $a=\frac{k_{1}}{m_{1}}\frac{k_{2}}{m_{2}},b=\frac{k_{1}}{m_{1}}+\frac{k_{2}}{m_{2}}+\frac{k_{2}}{m_{1}}$.

Here, the equivalent spring elasticity coefficients and the equivalent masses are

(3) $$k_{1}=\frac{\rho_{0}c_{0}^{2}}{V_{1}}$$

(4) $$k_{2}=\frac{\rho_{0}c_{0}^{2}}{V_{2}}$$

(5) $$m_{1}=\frac{\rho_{0}J_{1}}{S_{1}}$$

(6) $$m_{2}=\frac{\rho_{0}J_{2}}{S_{2}}$$

Here, $\rho_{0}$ is the density of the air, $c_{0}$ is the velocity of sound in air, *D*_1_ and *D*_2_ correspond to the diameters of the inner and outer holes, and *S* indicates the cross-sectional area of the holes, whose sizes are

(7) $$S_{1}=\pi {\left(\frac{D_{1}}{2}\right)}^{2}$$

(8) $$S_{2}=\pi {\left(\frac{D_{2}}{2}\right)}^{2}$$

Here, *J* is the effective length of the holes, and *t* is the wall thickness, whose sizes are

(9) $$J_{1}=t+0.305D_{1}+\frac{4}{3\pi }D_{1}$$

(10) $$J_{2}=t+0.305D_{2}+\frac{4}{3\pi }D_{2}$$

and the volumes of the chambers are

(11) $$V_{1}={\left(L_{1}-2t\right)}^{3}$$

(12) $$V_{2}={\left(L_{2}-2t\right)}^{3}$$

Similarly, the NSHT model with a negative effective mass density can be equated to a parallel double-spring oscillator model, as illustrated in Figure 1c. According to the acoustic force analogy theory, the resonant frequency of two resonant tubes with different tube lengths can be determined by considering the tube mouth as an acoustic spring and the bottom of the tube as an acoustic mass.

Moreover, due to the slight difference between the actual tube length and the resonant tube length, it is essential to incorporate the flange correction and coupling term into the final resonance frequency calculation. The resulting equation is as follows:

(13) $$f_{2}=\frac{c}{4*\left(\sqrt{{l_{2}}^{2}-\left({r_{2}}^{2}-{\left(r_{1}+t\right)}^{2}\right)}+0.305*2*\sqrt{{r_{2}}^{2}-{\left(r_{1}+t\right)}^{2}}\right)}\cdot e^{-k_{2}\cdot \left(\sqrt{{r_{2}}^{2}-{\left(r_{1}+t\right)}^{2}}+\delta \right)}$$

(14) $$f_{1}=\frac{c}{4*\left(\sqrt{-{r_{1}}^{2}+{l_{1}}^{2}}+0.305*2*r_{1}\right)}\cdot e^{-k_{1}\cdot \left(d_{1}+\delta \right)}$$

where *r*_1_ and *r*_2_ correspond to the inner and outer tube radii, respectively, *k_i_* is a constant of proportionality, and *δ* is a correction coefficient related to the coupling effect. See Refs. [31,32] for the specific theoretical derivation.

## 3. Results and Discussion

### 3.1. Resonant Metasurfaces Based on Single-Layer Structures

**Figure 2:**
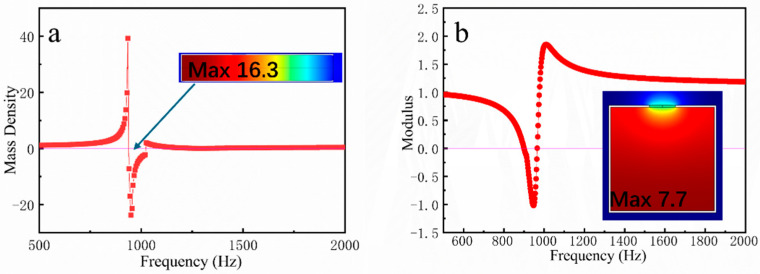
(**a**) Effective mass density curve of SHT and (**b**) effective modulus curve of SHC.

Figure 2 shows the characteristic curves of equivalent modulus versus equivalent mass for a single-layer negative effective parameter structure. At resonance frequency, acoustic energy accumulates within the cavity, where the air resonates at a specific frequency independent of the external sound field. Under these conditions, the normalized sound pressure level reaches 16.3 (SI units) in the SHT and 7.7 in the SHC. Consequently, the SHT demonstrates a greater magnitude of negative effective parameters compared to the SHC. The transmission spectra at resonance exhibit distinct absorption peaks featuring phase inversion and peak position shifts, which provides a solid theoretical basis for resonant metasurface development.

**Figure 3:**
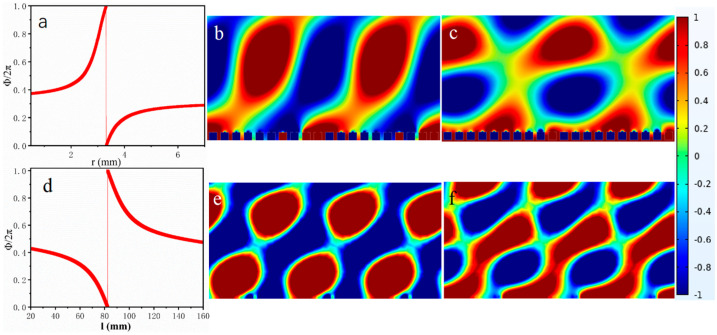
(**a**) Phase variation versus split-hole diameter in SHC at the frequency of 1000 Hz; anomalous reflected acoustic pressure field distributions plot for SHC at (**b**) 1000 Hz and (**c**) 1150 Hz; (**d**) phase variation versus tube length in SHT at 1000 Hz; anomalous reflected acoustic pressure field distribution for SHT at the different frequencies of (**e**) 1000 Hz and (**f**) 1150 Hz.

The metasurfaces were designed using two distinct configurations: an SHC exhibiting negative effective modulus and a single-layer SHT structure demonstrating negative effective mass properties. As shown in Figure 3, a complete 2π phase variation is achieved by adjusting either the hole radius (*r*) of SHC or the tube length (*l*) of SHT at the designated center frequency of 1000 Hz. Through optimization of the structural parameters (25 mm subunit spacing and *n* = 8 subunits), the metasurfaces with a phase gradient of π/100 were fabricated to enable precise acoustic wavefront modulation. Comparative analysis reveals that SHC-based metasurfaces show inferior wavefront modulation performance at two randomly selected frequencies (Figure 3) relative to their SHT counterparts. Given that the magnitude of the negative characteristic is indicative of the energy of the resonance unit at the frequency, it may be posited that the magnitude of the negative characteristic is directly proportional to the shaping effect of the final metasurfaces. That is to say, enhanced acoustic modulation efficiency and superior wavefront control are achieved when the absolute value of the negative parameter increases, as this corresponds to greater energy participation.

**Figure 4:**
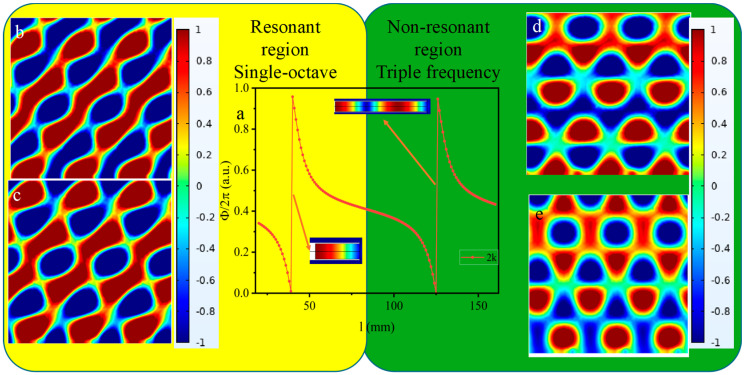
(**a**) Phase variation versus tube lengths in SHT at 2000 Hz; anomalous reflected acoustic pressure field distribution for SHT at (**b**) 2000 Hz, (**c**) 2200 Hz in the resonance region, and (**d**) 2000 Hz and (**e**) 2200 Hz in the non-resonance region.

Building upon the established findings that SHT exhibits enhanced acoustic performance in the resonant region, we extended our investigation to the non-resonant regime, with specific focus on metasurface (AMS) design at triple frequency (2 kHz) [38]. As shown in Figure 4, the AMS was implemented in this frequency band by utilizing the abrupt phase transition zones in both resonant and non-resonant domains. The phase gradient and period were configured as π/100 rad/mm and 5 mm, respectively. These simulation results reveal that the resonant-region-designed AMSs achieve substantially broader anomalous reflection bandwidths and superior wavefront shaping capabilities compared to their non-resonant-region counterparts. In summary, the imaging effect of a metasurface without “negative effective mass” relying solely on structural phase modulation is suboptimal. The metasurface exhibits evident limitations in beam manipulation and high-efficiency energy focusing, attributable to the configuration of the unit without “negative effective mass density”, which hinders effective modulation of the local resonance energy accumulation. These results substantiate our initial hypothesis that structures possessing stronger negative effective parameter characteristics enable more effective acoustic wave manipulation in metasurface implementations.

To comprehensively validate this conclusion, comparative metasurface designs were implemented using a single-layer cube and a single-layer hollow circular tube in the 2 kHz and 3 kHz bands, respectively. The experimental results confirm that the final wavefront shape of the metasurfaces constructed with the SHT, which exhibits superior acoustic performance, is markedly superior to that of the SHC in both frequency bands. These findings substantiate the critical importance of negative effective parameters in acoustic metasurface design.

Notably, our experimental observations revealed that although both tube length and diameter critically influence the resonance frequency, diameter variation unexpectedly exhibited negligible phase modulation effects. This empirical evidence suggests that it is not feasible to directly design the metasurfaces by modifying the tube diameter. This finding indicates that more precise control of the structural parameters, particularly those that have a notable impact on the phase shift, is essential for the design of metasurfaces to achieve the desired acoustic modulation effect.

In summary, this study advances the understanding of SHT’s acoustic characteristics under both resonant and non-resonant conditions while providing essential theoretical foundations and practical guidelines for metasurface engineering. Future investigations should explore alternative structural materials with superior acoustic properties to optimize metasurface design methodologies and facilitate advancements in acoustic wave modulation technologies.

### 3.2. Resonant Metasurfaces Based on Tandem Nested Bilayers

**Figure 5:**
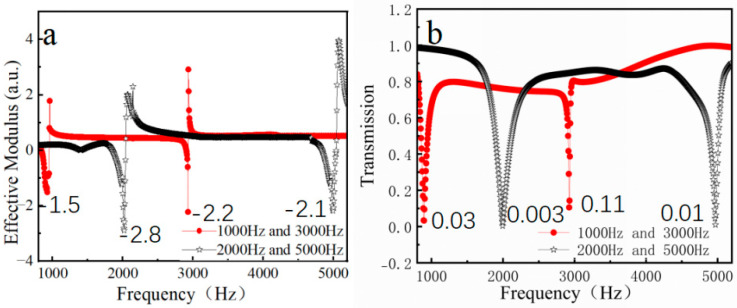
(**a**) Effective modulus curve and (**b**) transmission curve of NSHC.

Based on the findings presented in Section 2, the NSHC structure demonstrates significant potential for developing tandem nested bilayer structure metasurfaces with negative effective parameters. This potential stems primarily from the strong coupling observed between its dual resonant frequencies. The coupling mechanism ensures that modifications to one resonant state induce corresponding adjustments in the other resonant state. Therefore, the design process necessitates a simultaneous tuning approach. Specifically, both hole parameters of the bilayer cube structure must be adjusted concurrently to accurately control the phases at two distinct frequencies, thereby constructing the desired metasurfaces. Through strategic integration of these frequency-specific metasurfaces, the system achieves simultaneous acoustic wave modulation in either the 1 kHz and 3 kHz or 2 kHz and 5 kHz frequency bands. As shown in Figure 5, the two resonant frequencies have similar negative effective parameter sizes or transmittances, indicating the same regulation effect in tandem configurations.

**Figure 6:**
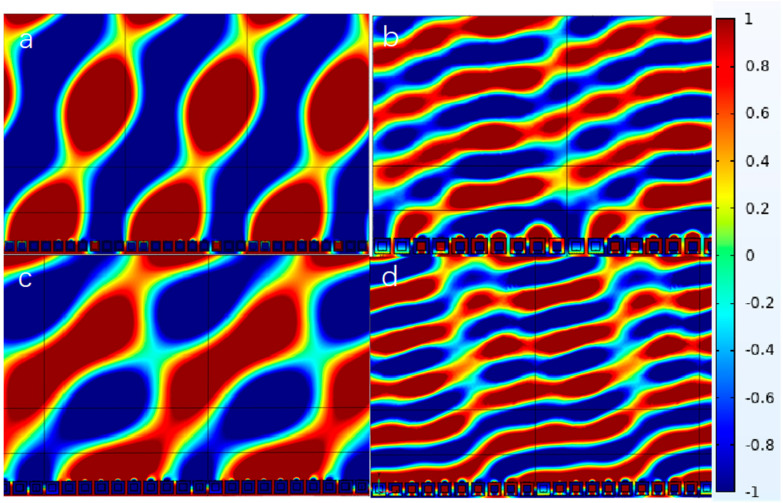
Reflected acoustic pressure field distribution of the NSHC-integrated metasurface at distinct frequencies: (**a**) 1000 Hz (low frequency), (**b**) 3000 Hz with phase gradient π/180 rad/mm, (**c**) 2000 Hz (low frequency), and (**d**) 5000 Hz with phase gradient π/120 rad/mm.

As illustrated in Figure 6, the metasurface design operating at dual-frequency combinations (1000/3000 Hz or 2000/5000 Hz) has been successfully implemented using the NSHC structure via a combined modulation approach. The waveform control exhibits relatively continuous characteristics across both frequency bands. This integrated strategy allows simultaneous acoustic wave manipulation in two distinct frequency ranges while maintaining identical phase gradients. However, the strong inter-element coupling in NSHCs limits design flexibility compared to the NSHT configuration. Specifically, the coupling effect restricts phase gradient resolution to a uniform value across the entire metasurface, unlike the NSHT case which permits finer gradient tuning (detailed comparison will be presented in Section 3.3). Additionally, the conventional layered architecture combining outer low-frequency and inner high-frequency components inherently restricts design diversity in dual-band metasurfaces.

To achieve dual-band modulation, the volume ratio between the inner layer and the interlayer of an NSHC must be strictly controlled within 0.15–1.0. This requirement ensures that the structure can accumulate sufficient energy for simultaneous low- and high-frequency modulation. Although exhibiting reduced tuning flexibility compared to the NSHT configuration, the NSHC structure demonstrates distinctive advantages, particularly in maintaining smaller discrepancies in negative effective parameters in both frequency bands. These characteristics suggest that NSHC-based metasurfaces may achieve enhanced imaging performance, particularly in high-frequency regimes, owing to their superior acoustic modulation capabilities.

In summary, the NSHC structure provides innovative perspectives and design methodologies for dual-band acoustic metasurface engineering. The structure’s remarkable wave manipulation capabilities position it as a viable solution for targeted applications, notwithstanding existing design constraints. Subsequent investigations should focus on structural optimization strategies to overcome current limitations and enable enhanced dual-frequency modulation performance with improved flexibility and efficiency.

### 3.3. Resonant Metasurfaces Based on Parallel Nested Double-Layer Structures

**Figure 7:**
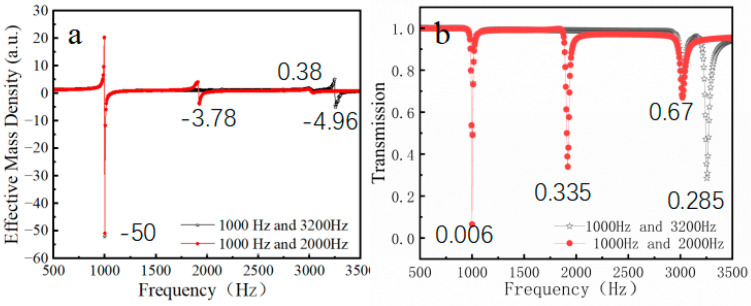
(**a**) Effective mass density curve and (**b**) transmission curve of NSHT.

From the theoretical derivation and equivalent model presented in Section 2, it can be observed that the two resonance frequencies of the NSHT are independent of each other. Additionally, the tunability is relatively high, as evidenced by the references cited in [39] (see Figure 7 below). A circular tube with independent tunability can be employed in the design of a metasurface using the method of separate tuning. The absolute value of the negative effective mass parameter is maximized for tubes operating at a lower frequency, and this value increases proportionally with frequency elevation. Furthermore, the negative effective parameter diminishes with rising frequency, whereas the effective parameter at the triple frequency is merely 0.38, failing to attain a negative value. This explains why metasurfaces formed by multiple frequencies exhibit significantly inferior performance compared to those at the fundamental frequency, as previously discussed. As shown in Figure 7, NSHT demonstrates lower transmittance and larger negative effective parameters (reaching up to 50) at low frequencies, indicating enhanced controllability in this regime. Consequently, metasurfaces can be designed with distinct phase gradients in both frequency bands.

**Figure 8:**
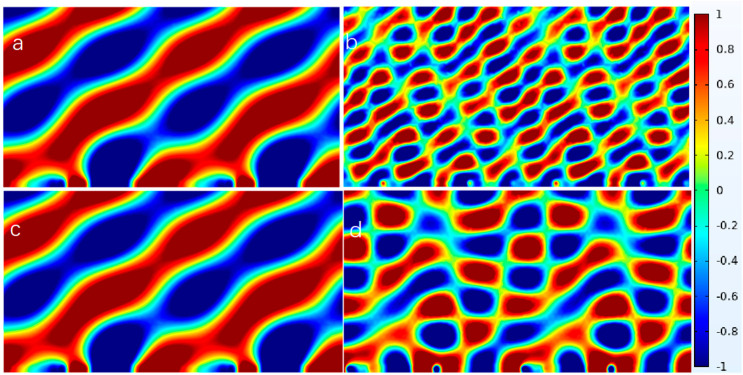
Reflected acoustic pressure field distribution of the metasurface with NSHT at different frequencies: (**a**) 1000 Hz (π/245 rad/mm), (**b**) 3200 Hz (π/140 rad/mm), (**c**) 1000 Hz (π/245 rad/mm), and (**d**) 2000 Hz (π/245 rad/mm).

Figure 8 illustrates the successful design of reflective metasurfaces at frequencies of 1000 Hz, 2000 Hz, and 3200 Hz, achieved through independent structural modulation of the NSHT. This design strategy enables effective sound wave modulation in dual-frequency bands (1000 Hz/2000 Hz or 1000 Hz/3200 Hz) via metasurface combinations. The NSHT exhibits identical negative effective parameters at low frequencies, resulting in equivalent waveform control effects. Notably, the negative effective parameter at 3000 Hz exceeds that at 2000 Hz, corresponding to marginally superior metasurface waveform control performance at the higher frequency. In the double-layer structure, both inner and outer circular tubes demonstrate functionally analogous characteristics, with equivalent contributions to acoustic wave modulation across low- and high-frequency ranges. This confirms that the resultant acoustic effect remains unaffected by the specific layer assignment as either a high- or low-frequency modulation unit, establishing complete functional independence between layers.

It is important to note that although the current design has achieved relative independence of the inner and outer layers, the significant difference in energy accumulation between the tubes at different frequencies results in mutual coupling between the two frequency bands, as illustrated in Figure 8. This phenomenon is more pronounced when the negative effective parameters of the second frequency band are lower, indicating that an increase in the negative effective parameters of the first frequency band enhances its coupling effect. However, this improvement simultaneously degrades the coupling effect of the second frequency band, which exhibits smaller negative effective parameters. The influence of the first-stage frequency diminishes due to its negative effective parameters. Furthermore, the independence of the inner and outer layers may be further compromised as the number of nested layers increases. Specifically, introducing additional acoustic interactions through more nested layers may lead to the emergence of minor noise or interference phenomena that would otherwise have negligible impact on the acoustic field distribution. These effects may become significant, ultimately degrading the overall performance of the metasurfaces.

It is therefore essential that future designs consider multiple factors, including the number of nested layers, structural parameters, and phase gradients, to maintain the excellent acoustic modulation capability of the metasurface while increasing its functional complexity. Furthermore, additional research is required to optimize the structural design, improve the modulation strategy, and enhance the material properties to boost the performance of dual-band and multi-band metasurfaces.

## 4. Conclusions

This work investigates the influence of negative effective parameters of resonant structure units near the central frequency bands on the imaging effect of this type of resonant metasurfaces. The results demonstrate a direct positive correlation between the imaging effect of single-layer resonant metasurfaces with a single frequency band and the absolute value of their negative effective parameter, which stems from enhanced resonant energy accumulation due to the increased negative effective parameter value, consequently improving imaging performance. For these nested resonance metasurfaces with dual bands, the imaging effect is significantly influenced by the magnitude of the negative effective parameter difference between two distinct frequency bands. Minimizing this difference optimizes dual-frequency imaging performance. Furthermore, metasurfaces with tandem-type structure unit designs exhibit reduced discrepancies in negative effective parameter values, thereby enhancing imaging performance. The parallel-type structure unit metasurfaces offer greater flexibility and variability in acoustic modulation effects. Therefore, this work provides valuable optimization strategies and practical guidance for achieving effective and stable acoustic modulation in resonant metasurfaces.

## Data Availability

The original contributions presented in this study are included in the article. Further inquiries can be directed to the corresponding authors.

## Abbreviations

The following abbreviations are used in this manuscript:

SHC	Split Hollow Cuboid
SHT	Single-Opening Hollow Tube
NSHC	Nested Split Hollow Cuboid
NSHT	Nested Single-Opening Hollow Tube
AMS	Acoustic Metasurface
PLA	Polylactic Acid
